# Advances in using PARP inhibitors to treat cancer

**DOI:** 10.1186/1741-7015-10-25

**Published:** 2012-03-09

**Authors:** Shivaani Kummar, Alice Chen, Ralph E Parchment, Robert J Kinders, Jay Ji, Joseph E Tomaszewski, James H Doroshow

**Affiliations:** 1 Division of Cancer Treatment and Diagnosis, Bldg. 31, Room 3A-44, 31 Center Drive, National Cancer Institute, Bethesda, MD, 20892, USA; 2 Applied/Developmental Research Directorate, Science Applications International Corporation-Frederick, Inc., Bldg. 431, 1050 Boyles St., National Cancer Institute at Frederick, Frederick, MD, 21702 USA; 3 Center for Cancer Research, Bldg. 37, Room 1052, 37 Convent Drive, National Cancer Institute, Bethesda, MD, 20892 USA

**Keywords:** synthetic lethality, DNA repair, PARP clinical trials

## Abstract

The poly (ADP-ribose) polymerase (PARP) family of enzymes plays a critical role in the maintenance of DNA integrity as part of the base excision pathway of DNA repair. PARP1 is overexpressed in a variety of cancers, and its expression has been associated with overall prognosis in cancer, especially breast cancer. A series of new therapeutic agents that are potent inhibitors of the PARP1 and PARP2 isoforms have demonstrated important clinical activity in patients with breast or ovarian cancers that are caused by mutations in either the *BRCA1 *or *2 *genes. Results from such studies may define a new therapeutic paradigm, wherein simultaneous loss of the capacity to repair DNA damage may have antitumor activity in itself, as well as enhance the antineoplastic potential of cytotoxic chemotherapeutic agents.

## Background

Environmental exposures and cell replication result in DNA damage that is repaired by a variety of mechanisms, including base excision repair (BER), mismatch repair (MMR), nucleotide excision repair (NER), single strand annealing (SSA), homologous recombination (HR), and nonhomologous end joining (NHEJ) [1]. Poly (ADP-ribose) polymerases (PARPs) are a family of proteins involved in DNA repair that utilize the BER pathway [2] and share enzymatic and scaffolding properties. PARP1 and PARP2 are the best studied members of this family of enzymes. PARP1 has three domains that are responsible for DNA-binding, automodification, and catalysis. DNA cleavage results in the recruitment and binding of PARP1 to the site of damage, with an increase in its catalytic activity, and the formation of long, branched, poly (ADP-ribose) (PAR) chains. PAR has a net negative charge that promotes recruitment of DNA repair proteins involved in the BER pathway to the site of DNA damage, and facilitates removal of PARP1 from damage sites, allowing access to other repair proteins. Apart from its role in BER, PARP1 has been implicated in the HR and NHEJ pathways, suggesting a broader role for this enzyme family in the overall DNA repair process.

PARPs were initially identified in 1963; the potential for PARP inhibition to enhance DNA damage caused by cytotoxic chemotherapy was first considered in 1980 [3,4]. PARP1 is overexpressed in a variety of cancers and its expression has been associated with overall prognosis in cancer, especially breast cancer [5]. PARP inhibitors in clinical development mimic the nicotinamide moiety of nicotinamide adenine dinucleotide, and bind to the enzyme's catalytic domain, inhibiting automodification and subsequent release of the enzyme from the site of DNA damage. In so doing, PARP inhibitors also prevent access of other repair proteins to the site of DNA cleavage.

Several PARP inhibitors are in clinical development (see Additional Files 1 and 2); as a whole, these agents have generated considerable interest because of their potential clinical activity for patients whose tumors harbor defects in the HR pathway [6-8]. Although several of these drugs have been shown to inhibit PARP *in vivo*, their spectrum of activity and effects on DNA repair pathways make them distinct. This review summarizes current insights into the mechanism of action, recent clinical trials, and potential next steps in the evaluation of this promising class of anti-cancer drugs.

## Mechanism of action and pharmacology of PARP inhibitors

A number of PARP inhibitors are under clinical development: rucaparib (CO-338; AG014699, PF-0367338; oral/IV), iniparib (BSI-201), olaparib (AZD-2281; oral), veliparib (ABT-888; oral), MK-4827, BMN-673, CEP-9722 (oral) and E7016 (GPI 21016, oral). The loss of BER capacity produced by PARP inhibition has prompted the evaluation of these drugs as potential enhancers of DNA damaging cytotoxic chemotherapeutic agents such as alkylating agents (for example, platinum, cyclophosphamide) and topoisomerase 1 inhibitors (for example, camptothecin analogs) [9]. However, recent studies strongly suggest that, unlike the other drugs, the mechanism of action of iniparib is unclear and is probably not related to PARP inhibition *per se *[10].

PARP inhibition enhances the therapeutic index of cytotoxic chemotherapy only if DNA damage is selectively increased in tumor compared to normal tissues, such as the gastrointestinal mucosa or bone marrow. The opportunity to achieve selectivity in tumor cell killing with these agents would, therefore, be improved in tumors that already harbor DNA repair defects. Simultaneous dysfunction of two DNA damage repair (DDR) pathways, termed 'synthetic lethality', decreases the ability of tumor cells to withstand the DNA damage produced during normal cellular replication [8]. Duplication of this phenomenon pharmacologically is possible in tumors harboring somatic or germline defects in a non-BER pathway of DDR by treating with a PARP inhibitor so that BER and non-BER pathways are blocked simultaneously. Clinical development programs are testing this idea directly in settings where the HR pathway is compromised, for example, with PARP inhibitor monotherapy for tumors with *BRCA1/2 *defects. This could also be extended to include treatment of tumors with defects in other HR pathway proteins. For instance, PTEN-deficient cells have been shown to be sensitive to PARP inhibition, due to the role of PTEN in the expression of RAD51 [11]. One question for the further development of PARP inhibitors is whether they can effectively enhance DNA damage, in the presence of DNA damaging agents, in tumors that lack an intrinsic defect in DDR.

New data are emerging on the myriad of effects of PARP on DNA repair and other pathways (see Additional File 3). PARP has also been implicated in DNA repair by recruiting mitotic recombination 11 (MRE11) and ataxia telangiectasia-mutated (ATM) to DNA DSBs; effects on BRCA 1 and RAD 51 expression via repressive E2F4 and p130 complexes; interaction with the DNA protein kinase (DNA PK) complex [Ku70, Ku80, DNA PK] involved in NHEJ of DSBs; and the epigenetic regulation of chromatin structure [2,12-15]. Recent reports evaluate the role of PARP in BER and its interaction with DNA single strand break intermediate forms. A differential effect of repair of SSBs has been demonstrated in the presence of PARP inhibitors as compared to PARP1 siRNA cells treated with the alkylating agent, dimethyl sulfate [16].

## Pharmacodynamics of PARP inhibitors on PARP1 and PARP2

Assays have been developed to quantify drug-induced inhibition of PARP enzymatic activity in patient specimens. The primary effect of PARP inhibitors changes two parameters, each of which could be used as a pharmacodynamic endpoint: decreased PARP1/2-specific activity and decreased production of PARP1/2 reaction products, which are poly-ADP-ribosylated macromolecules ('PARylated substances'). However, a major concern with any *ex vivo *enzymatic assay is the dilution of the extract with sample processing and enzyme assay buffers. Diluting a tissue sample also dilutes the concentration of the competitive PARP inhibitor that was present at the time of sample collection. Under conditions of linear enzyme kinetics, the measured enzymatic activity may be more an indication of the resulting drug concentration in each diluted sample, rather than a measure of enzymatic activity originally present in the tissue in the face of actual tissue concentrations of the drug.

On the other hand, measurement of PARylated substances produced by PARP1/2 activity reflects the balance between degradation of those PARylated molecules by an enzyme called poly (ADP-ribose) glycohydrolase (PARG), and production by PARP1/2. A sandwich immunoassay (IA) was developed and validated at the US National Cancer Institute to quantify the level of poly-ADP-ribosylated macromolecules from a calibration curve of poly-ADP-ribose standard ('PAR antigen') [17]. This PAR-IA was designed for the first-in-human use of veliparib, where the measurement of PARylated substances would be the primary objective of a Phase '0' clinical trial. Sufficient assay sensitivity was required to distinguish a 30% decrease in the PARP1/2 reaction product PAR, with a lower limit of quantitation sufficient to quantify a 90% drop in PAR relative to baseline in approximately 85% of paired mononuclear cell samples. Prior to the clinical trial, a fit-for-purpose study was conducted in mice harboring human tumor xenografts to model the proposed use of the PAR IA. The results proved that veliparib significantly decreased PAR levels from baseline by four to seven hours after a single oral dose - the timeframe planned for the clinical biopsies. The results of the subsequent first-in-human clinical trial confirmed the findings of the fit-for-purpose animal modeling studies [18].

The PAR IA has been used to define a reproducible response of PARP1/2 to veliparib in tumor biopsies and mononuclear cell samples from treated patients. In addition, the PAR IA has been used to confirm pharmacodynamic effects, similar to those observed with veliparib, produced by olaparib and MK-4827 in human tumor xenografts and human tumor cell lines *in vitro*. However, iniparib and its two major metabolites failed to cause any change in the level of PARylated substances in the model systems, as measured by the PAR-IA [10]. The apparent lack of a pharmacodynamic effect by iniparib on PARP1/2 is very different from the responses elicited by veliparib, olaparib, and MK-4827. This may explain the recently reported lack of an effect of iniparib on the efficacy and toxicity of combination chemotherapy for triple negative breast cancer (TNBC) patients in a properly powered Phase III clinical trial [19].

## Clinical experience with PARP inhibitors

PARP inhibitors have been evaluated in clinical trials either as single agents, with an emphasis on patients carrying *BRCA *mutations, or in combination with DNA damaging therapies (see Additional Files 1 and 2). Olaparib has demonstrated single agent activity in breast or ovarian cancer patients with germline mutations in *BRCA1/2 *[20-22]; an over 40% response rate has been reported in patients with *BRCA *mutant ovarian cancer, especially in patients with platinum sensitive disease [23].

The Cancer Genome Atlas Research Project recently reported on the molecular aberrations in high grade serous ovarian adenocarcinoma, demonstrating a defect in the HR pathway in half of the 489 tumors analyzed [24]. These results suggest that ovarian cancer patients with sporadic abnormalities in the HR pathway impairing DNA repair might benefit from treatment with PARP inhibitors. Similar abnormalities in DNA repair pathways have been reported in primary peritoneal cancers, and in patients with TNBC, forming the basis for recent clinical trials that have explored the use of PARP inhibitors in such patient populations [25-28]. Tumor types with defects in other DNA repair pathways, such as tumors with microsatellite instability, may also be susceptible to inhibition of the BER pathway [29].

Despite the evaluation of PARP inhibition in a number of clinical trials, the degree and duration of inhibition required for optimal clinical benefit has yet to be established [18]. This has resulted in the continuation of studies that have explored higher PARP inhibitor doses, well beyond those demonstrated to result in near-complete inhibition of PARP activity in clinical tumor samples; the results of some of these trials, such as the ICEBERG study, have suggested a dose response for deriving clinical benefit from PARP inhibitors [21,22,30].

## Conclusions and outlook for the use of PARP inhibitors in the future

A major focus for the future clinical development of PARP inhibitors is to determine whether or not potentiating chemotherapy- or radiation-induced DNA damage in patients without known defects in DDR is either possible or fruitful. Enhancement of DNA damage by the addition of a PARP inhibitor to a topoisomerase I poison has been demonstrated in tumor biopsies and circulating tumor cells by measurement of γH2AX foci, a marker of DNA double strand breaks, in patients treated with veliparib and topotecan compared to those receiving topotecan alone [31]. However, the development of PARP inhibitors as chemopotentiating agents has been limited by an increase in observed toxicities, mainly myelosuppression, necessitating dose reductions of the cytotoxic chemotherapeutic agent and the PARP inhibitor [31,32]. This raises the question of whether administering the combination is more efficacious than administering full doses of the chemotherapeutic agent alone, as well as the need to design clinical trial strategies to improve the therapeutic index of these combinations. It seems likely that to optimize the use of PARP inhibitors in the future will require the development of predictive assays to determine the presence of unsuspected defects in DDR pathways in tumors. It also presents an opportunity to rationally develop combinations of PARP inhibitors with new classes of DNA repair inhibitors that are on the horizon, and classical cytotoxic agents [33].

## Abbreviations

ATM: ataxia telangiectasia-mutated; BER: base excision repair; DDR: DNA damage repair; DNA PK: DNA protein kinase; DSBs: double strand breaks; HR: homologous recombination; MMR: mismatch repair; MRE11: mitotic recombination 11; NER: nucleotide excision repair; NHEJ: non-homologous end joining; PARG: poly (ADP-ribose) glycohydrolase; PARP: poly (ADP-ribose) polymerase; TNBC: triple negative breast cancer

## Competing interests

The authors declare that they have no competing interests.

## Authors' contributions

SK, AC, REP, RK, and JHD jointly wrote the manuscript. All authors were involved in revising the manuscript, and gave final approval. All authors read and approved the final manuscript.

## Pre-publication history

The pre-publication history for this paper can be accessed here:

http://www.biomedcentral.com/1741-7015/10/25/prepub

## Supplementary Material

Additional File 1 **Early phase clinical trials with PARP inhibitors**. A table listing clinical trials of PARP inhibitors currently in development in early phase clinical trials. Click here for file

Additional File 2 **Clinical trials with PARP inhibitors in defined diseases**. A table listing clinical trials of PARP inhibitors currently in development in specified cancers. Click here for file

Additional File 3 **Structural and functional characteristics of PARP1. A**: Poly(ADP-ribose) polymerase 1 (PARP1) is shown with its DNA-binding (DBD), automodification (AD) and catalytic domains. The PARP signature sequence (yellow box within the catalytic domain) comprises the sequence most conserved among PARPs. Crucial residues for nicotinamide adenine dinucleotide (NAD+) binding (histidine; H and tyrosine; Y) and for polymerase activity (glutamic acid; E) are indicated. **B: **Consequences of PARP1 activation by DNA damage. Although not shown to simplify the scheme, PARP1 is active in a homodimeric form. PARP1 detects DNA damage through its DBD. This activates PARP1 to synthesize poly(ADP) ribose (pADPr; yellow beads) on acceptor proteins, including histones and PARP1. Owing to the dense negative charge of pADPr, PARP1 loses affinity for DNA, allowing the recruitment of repair proteins by pADPr to the damaged DNA (blue and purple circles). Poly(ADP-ribose) glycohydrolase (PARG) and possibly ADP-ribose hydrolase 3 (ARH3) hydrolyse pADPr into ADP-ribose molecules and free pADPr. ADP-ribose is further metabolized by the pyrophosphohydrolase NUDiX enzymes into AMP, raising AMP:ATP ratios, which in turn activate the metabolic sensor AMP-activated protein kinase (AMPK). NAD+ is replenished by the enzymatic conversion of nicotinamide into NAD+ at the expense of phosphoribosylpyrophosphate (PRPP) and ATP. Examples of proteins non-covalently (pADPr-binding proteins) or covalently poly(ADP-ribosyl)ated are shown with the functional consequences of modification. It is important to note that many potential protein acceptors of pADPr remain to be identified owing to the difficulty of purifying pADPr-binding proteins *in vivo*. PARP inhibitors prevent the synthesis of pADPr and hinder subsequent downstream repair processes, lengthening the lifetime of DNA lesions. ATM, ataxia telangiectasia-mutated; BER, base excision repair; BRCT, BRCA1 carboxy-terminal repeat motif; DNA-PKcs, DNA-protein kinase catalytic subunit; DSB, double-strand break; HR, homologous recombination; NHEJ, non-homologous end joining; NLS, nuclear localization signal; PPi, inorganic pyrophosphate; SSB, single-strand break; Zn, zinc finger. Reprinted by permission from Macmillan Publishers Ltd: Nature Reviews Cancer [2], copyright (2010). Click here for file
