# Supplementary material for: Advances in using PARP inhibitors to treat cancer
Source: BMC Med. 2012 Mar 9;10:25. doi: 10.1186/1741-7015-10-25 (PMC3312820; doi:10.1186/1741-7015-10-25)
Supplement: Additional File 1 — Early phase clinical trials with PARP inhibitors. A table listing clinical trials of PARP inhibitors currently in development in early phase clinical trials. [file 1741-7015-10-25-S1.PDF]

Early Phase Clinical Trials with PARP Inhibitors

Agent(s)	Histology and Reference*
**BMN-673**	
BMN 673	Advanced Solid Tumor [NCT01286987]
**CEP-9722**	
CEP 9722	Advanced Solid Tumor [NCT01311713]
CEP 9722 + Temozolomide	Advanced Solid Tumor [NCT00920595]
CEP 9722 + Gemcitabine + Cisplatin	Advanced Solid Tumor [NCT01345357]
**CO-338; Rucaparib (AG014699, PF-0367338)**	
Rucaparib (CO-338, AG014699, PF-01367338) + Carboplatin	Advanced Solid Tumor [NCT01009190]
**E7016 (GPI 21016)**	
E7016 + Temozolomide	Advanced Solid Tumor [NCT01127178]
**MK4827**	
MK4827 (Single Agent)	Advanced Solid Tumor (enriched for *BRCA*+ histologies) [1]
MK4827 + Carboplatin; Carboplatin + Paclitaxel; Carboplatin + PLD (Terminated)	Advanced Solid Tumor [NCT01110603]
MK4827 + PLD	Advanced Solid Tumor, Ovarian Cancer [NCT01227941]
MK4827 + Temozolomide	Advanced Solid Tumor [NCT01294735]
**Olaparib (AZD2281)**	
Olaparib (Single Agent)	Advanced Solid Tumor (*BRCA 1/2+)* [2]
Olaparib + Cisplatin	Advanced Solid Tumor [NCT00782574]
Olaparib + Dacarbazine	Advanced Solid Tumor [3]
Olaparib + Liposomal Doxorubicin	Advanced Solid Tumor [NCT00819221]
Olaparib + Topotecan	Advanced Solid Tumor [4]
Olaparib + Cisplatin + Gemcitabine	Advanced Solid Tumor [5]
**Veliparib (ABT-888)**	
Veliparib (Single Agent)	Advanced Solid Tumor [6]
Veliparib + Cyclophosphamide	Advanced Solid Tumor [7]
Veliparib + Metronomic Cyclophosphamide	Advanced Solid Tumor [8]
Veliparib + Gemcitabine	Advanced Solid Tumor [NCT01154426]
Veliparib + Irinotecan	Advanced Solid Tumor [9]
Veliparib ± Mitomycin	Metastatic, Unresectable, or Recurrent Solid Tumor [10]

Agent(s)	Histology and Reference*
Veliparib + Temozolomide	Advanced Solid Tumor [NCT01193140, NCT00526617]
Veliparib + Topotecan	Advanced Solid Tumor [11]
Veliparib + Carboplatin + Gemcitabine	Advanced Solid Tumor [NCT01063816]
Veliparib + Carboplatin + Paclitaxel	Advanced Solid Tumor (enriched for *BRCA*+ histologies) [NCT00535119]
	Advanced Solid Tumor [NCT01281150]
	Advanced Solid Tumor (with liver or kidney problems) [NCT01366144
Veliparib + Cyclophosphamide + Doxorubicin	Adult Solid Tumor [12]
Veliparib + Dinaciclib ± Carboplatin	Advanced Solid Tumor (enriched for *BRCA1/2* histologies) [NCT01434316]
Veliparib + Oxaliplatin + Capecitabine	Advanced Solid Tumor [NCT01233505]

*Publication or ClinicalTrials.gov identifier.

Abbreviations: PLD, Pegylated Liposomal Doxorubicin.

Refrences

1. Schelman WR, Sandhu SK, Moreno Garcia V, Wilding G, Sun L, Toniatti C, Stroh M, Kreischer N, Carpenter CL, Molife LR, Kaye SB, de Bono JS, Wenham RM: **First-inhuman trial of a poly(ADP)-ribose polymerase (PARP) inhibitor MK-4827 in advanced cancer patients with antitumor activity in BRCA-deficient tumors and sporadic ovarian cancers (soc) [abstract].** *J Clin Oncol* 2011, **29**(Suppl 15):3102

2. Fong PC, Boss DS, Yap TA, Tutt A, Wu P, Mergui-Roelvink M, Mortimer P, Swaisland H, Lau A, O'Connor MJ, Ashworth A, Carmichael J, Kaye SB, Schellens JH, de Bono JS: **Inhibition of poly(ADP-ribose) polymerase in tumors from BRCA mutation carriers.** *N Engl J Med* 2009, **361:**123-134.

3. Khan OA, Gore M, Lorigan P, Stone J, Greystoke A, Burke W, Carmichael J, Watson AJ, McGown G, Thorncroft M, Margison GP, Califano R, Larkin J, Wellman S, Middleton MR: **A phase I study of the safety and tolerability of olaparib (AZD2281, KU0059436) and dacarbazine in patients with advanced solid tumours.** *Br J Cancer* 2011, **104:**750-755.

4. Samol J, Ranson M, Scott E, Macpherson E, Carmichael J, Thomas A, Cassidy J: **Safety and tolerability of the poly(ADP-ribose) polymerase (PARP) inhibitor, olaparib (AZD2281) in combination with topotecan for the treatment of patients with advanced solid tumors: a phase I study.** *Invest New Drugs*, in press.

5. Giaccone G, Rajan A, Kelly RJ, Gutierrez M, Kummar S, Yancey M, Ji JJ, Zhang Y, Parchment RE, Doroshow JH: **A phase I combination study of olaparib (AZD2281;KU-0059436) and cisplatin (C) plus gemcitabine (G) in adults with solid tumors [abstract].** *J Clin Oncol* 2010, **28**(Suppl 15):3027

6. Kummar S, Kinders R, Gutierrez ME, Rubinstein L, Parchment RE, Phillips LR, Ji J, Monks A, Low JA, Chen A, Murgo AJ, Collins J, Steinberg SM, Eliopoulos H, Giranda VL, Gordon G, Helman L, Wiltrout R, Tomaszewski JE, Doroshow JH: **Phase 0 clinical trial of the poly (ADP-ribose) polymerase inhibitor ABT-888 in patients with advanced malignancies.** *J Clin Oncol* 2009, **27:**2705-2711.

7. Tan AR, Gibbon D, Stein MN, Moss RA, Karantza V, Lin H, Gounder M, Chen AP, Egorin MJ, DiPaola RS: **Preliminary results of a phase I trial of ABT-888, a poly(ADP-ribose) polymerase (PARP) inhibitor, in combination with cyclophosphamide [abstract].** *J Clin Oncol* 2010, **28**(Suppl 15):3000

8. Kummar S, Chen AP, Ji JJ, Allen D, Egorin MJ, Gandara DR, Lenz H, Morgan R, Newman EM, Doroshow JH: **A phase I study of ABT-888 (A) in combination with metronomic cyclophosphamide (C) in adults with refractory solid tumors and lymphomas [abstract].** *J Clin Oncol* 2010, **28**(Suppl 15):2605

9. LoRusso P, Ji JJ, Li J, Heilbrun LK, Shapiro G, Sausville EA, Boerner SA, Smith DW, Pilat MJ, Zhang J, Chen AP, Nechiporchik N, Parchment RE: **Phase I study of the safety, pharmacokinetics (PK), and pharmacodynamics (PD) of the poly(ADP-ribose) polymerase (PARP) inhibitor veliparib (ABT-888; V) in combination with irinotecan (CPT-11; Ir) in patients (pts) with advanced solid tumors [abstract].** *J Clin Oncol* 2011, **29**(Suppl 15):3000

10. Zhao W, Duan W, Leon ME, Chen AP, Sofletea G, Thurmond J, Ramaswamy B, O'Malley D, Bekaii-Saab TS, Calero MA: **Targeting fanconi anemia (FA) repair pathway deficiency for treatment with PARP inhibitors [abstract].** *J Clin Oncol* 2010, **28**(Suppl 15):TPS168

11. Kummar S, Chen A, Ji J, Zhang Y, Reid JM, Ames M, Jia L, Weil M, Speranza G, Murgo AJ, Kinders R, Wang L, Parchment RE, Carter J, Stotler H, Rubinstein L, Hollingshead M, Melillo G, Pommier Y, Bonner W, Tomaszewski JE, Doroshow JH: **Phase I study of ABT-888, a PARP inhibitor, in combination with topotecan hydrochloride in adults with refractory solid tumors and lymphomas.** *Cancer Res* 2011, **71:**5626-5634.

12. Tan AR, Toppmeyer D, Stein MN, Moss RA, Gounder M, Lindquist DC, Ji JJ, Chen AP, Egorin MJ, Kiesel B, Beumer JH, DiPaola RS: **Phase I trial of veliparib, (ABT-888), a poly(ADP-ribose) polymerase (PARP) inhibitor, in combination with doxorubicin and cyclophosphamide in breast cancer and other solid tumors [abstract].** *ASCO Meeting Abstracts* 2011, **29**(Suppl 15):3041
