# Supplementary material for: Advances in using PARP inhibitors to treat cancer
Source: BMC Med. 2012 Mar 9;10:25. doi: 10.1186/1741-7015-10-25 (PMC3312820; doi:10.1186/1741-7015-10-25)
Supplement: Additional File 2 — Clinical trials with PARP inhibitors in defined diseases. A table listing clinical trials of PARP inhibitors currently in development in specified cancers. [file 1741-7015-10-25-S2.PDF]

## Clinical Trials with PARP Inhibitors in Defined Diseases

| Agent(s) | Histology and Reference* |
|---|---|
| **BMN-673** | |
| BMN 673 (Lt-673) (Single Agent) | Hematological Malignancies [NCT01399840] |
| **CO-338; Rucaparib (AG014699, PF-0367338)** | |
| Rucaparib (CO-338) | Breast or Ovarian Cancer (enriched for *BRCA* histologies) [1] |
| Rucaparib (CO-338) Oral | Breast Cancer or other Solid Tumor (enriched for *BRCA* histologies) [NCT01482715] |
| **Olaparib (AZD2281)** | |
| Olaparib (Single Agent) | Ovarian, Primary Peritoneal, and Fallopian Tube Cancer (*BRCA 1/2+*) [2] |
| | Ovarian Cancer (*BRCA 1/2+*) [3, 4] |
| | TNBC or High-Grade Serous and/or Undifferentiated Ovarian Cancer [5] |
| | Metastatic Colorectal Cancer (stratified by microsatellite status) [6] |
| Olaparib + Carboplatin | Breast or Ovarian Cancer (*BRCA+*) [7] |
| | Refractory Gynecologic Cancers; i.e. Breast, Ovarian, Fallopian, Primary Peritoneal, Uterine, and Cervical Cancer, or Malignant Mixed Mullerian Tumor [NCT01237067] |
| Olaparib + Cediranib | Recurrent Ovarian or Metastatic TNBC [8] |
| Olaparib versus Liposomal Doxorubicin | Ovarian (*BRCA+*) or Breast (*BRCA+*) Cancer (Failed Prior Platinum) [9] |
| ICEBERG 3 | |
| Olaparib + Gemcitabine | Advanced Pancreatic Cancer [NCT00515866] |
| Olaparib + Irinotecan | Advanced Colorectal Cancer [NCT00535353] |
| Olaparib + Paclitaxel; Olaparib + Carboplatin; Olaparib + Paclitaxel/Carboplatin | Advanced TNBC or Ovarian Cancer [NCT00516724] |
| Olaparib + Temozolomide | Advanced GBM [NCT01390571] |
| IC or ICM ± Olaparib | Advanced Pancreatic Cancer [NCT01296763] |
| Paclitaxel ± Olaparib | Advanced Gastric Cancer [NCT01063517] |
| Paclitaxel/Carboplatin ± Olaparib | Advanced Ovarian Cancer [NCT01081951] |
| **Veliparib (ABT-888)** | |
| Veliparib (Single Agent) | Tumors (enriched for *BRCA* or with *BRCA*ness features) |
| | BRCA+ ovarian cancer [NCT01472783] |

| Agent(s) | Histology and Reference* |
|---|---|
| Veliparib + Carboplatin | Breast Cancer [NCT01042379] |
| *Ispy2:* Neoadjuvant (Single Agent) | |
| Veliparib + Carboplatin | Breast Cancer (*HER2-)* [NCT01251874], [10] |
| Veliparib ± Carboplatin | Stage III or Stage IV Breast Cancer (*BRCA1/2*) [NCT01149083] |
| Veliparib + Metronomic Cyclophosphamide | TNBC, Ovarian (*BRCA*+), and Low Grade NHL [NCT01306032] |
| Veliparib + PLD | Recurrent Ovarian Cancer, Fallopian Tube Cancer, or Primary Peritoneal Cancer or Metastatic Breast Cancer [NCT01145430] |
| Veliparib + FOLFIRI | Colorectal Cancer [NCT01123876] |
| Veliparib + low-dose fractionated whole abdominal radiation therapy | Peritoneal Carcinomatosis [NCT01264432] |
| Veliparib ± Mitomycin | Metastatic, Unresectable, or Recurrent Solid Tumor [11] |
| Veliparib + Temozolomide | Metastatic Breast Cancer [46]; Melanoma [12]; Hepatocellular Carcinoma [NCT01205828]; Solid Tumors, Metastatic Melanoma, Breast Cancer (*BRCA*-), Ovarian, Primary Peritoneal, or Fallopian Tube Cancer, and Hepatocellular Carcinoma [NCT00526617]; Children With Recurrent/Refractory Central Nervous System Tumors [NCT00994071]; Colorectal Cancer [13]; Recurrent GBM [NCT01026493]; Metastatic Prostate Cancer [ NCT01085422]; Acute Leukemia [NCT01139970] |
| Veliparib + Temozolomide Versus PLD Alone | Ovarian Cancer [NCT01113957] |
| Veliparib + Topotecan | Relapsed or Refractory Ovarian [NCT01012817] |
| Veliparib + 5-FU + Oxaliplatin | Pancreatic Cancer [14] |
| Veliparib + Bendamustine + Rituximab | Lymphoma, Multiple Myeloma, or Adult Solid Tumor (NCT01326702) |
| Veliparib + Topotecan + Filgrastim or Pegfilgrastim | Persistent or Recurrent Cervical Cancer [NCT01266447] |
| Veliparib + Cisplatin + Gemcitabine | Advanced Biliary, Pancreatic, Urothelial, or NSCLC [NCT01282333] |
| Veliparib + Cisplatin + Paclitaxel | Advanced, Persistent, or Recurrent Cervical Cancer [NCT01281852] |
| Veliparib + Cisplatin + Vinorelbine | TNBC, Breast Cancer (*BRCA*) [NCT01104259] |
| Veliparib + Temozolomide + XRT | Newly Diagnosed GBM [NCT00770471] |
| Veliparib + Topotecan ± Carboplatin | Relapsed or Refractory Acute Leukemia, High-Risk Myelodysplasia, or Aggressive Myeloproliferative Disorders [NCT00588991] |
| Veliparib + Carboplatin + Paclitaxel + Bevacizumab | Newly Diagnosed Stage II, III, or IV Ovarian Cancer [NCT00989651] |
| Veliparib + Carboplatin + Paclitaxel + XRT | NSCLC Stage III [NCT01386385] |

| Agent(s) | Histology and Reference* |
|---|---|
| Cyclophosphamide ± Veliparib | Breast Cancer (*ER*+) [15] |

*Publication or ClinicalTrials.gov identifier.
Abbreviations: 5-FU, Fluorouracil; ER, estrogen receptor; FOLFIRI, Leucovorin, Fluorouracil, and Camptosar; GBM, Glioblastoma; IC, Irinotecan and Cisplatin; ICM, Irinotecan, Cisplatin, and Mitomycin; NSCLC, Non-small Cell Lung Cancer; NHL, Non-Hodgkin's Lymphoma; PLD, Pegylated Liposomal Doxorubicin; TNBC, Triple-Negative Breast Cancer; XRT, radiotherapy.

## Refrences


1. Drew Y, Ledermann JA, Jones A, Hall G, Jayson GC, Highley M, Rea D, Glasspool RM, Halford SER, Crosswell G, Colebrook S, Boddy AV, Curtin NJ, Plummer ER: **Phase II trial of the poly(ADP-ribose) polymerase (PARP) inhibitor AG-014699 in BRCA 1 and 2-mutated, advanced ovarian and/or locally advanced or metastatic breast cancer [abstract].** *J Clin Oncol* 2011, **29**(Suppl 15):3104

2. Fong PC, Yap TA, Boss DS, Carden CP, Mergui-Roelvink M, Gourley C, De GJ, Lubinski J, Shanley S, Messiou C, A'Hern R, Tutt A, Ashworth A, Stone J, Carmichael J, Schellens JH, de Bono JS, Kaye SB: **Poly(ADP)-ribose polymerase inhibition: frequent durable responses in BRCA carrier ovarian cancer correlating with platinum-free interval.** *J Clin Oncol* 2010, **28:**2512-2519.

3. Audeh MW, Carmichael J, Penson RT, Friedlander M, Powell B, Bell-McGuinn KM, Scott C, Weitzel JN, Oaknin A, Loman N, Lu K, Schmutzler RK, Matulonis U, Wickens M, Tutt A: **Oral poly(ADP-ribose) polymerase inhibitor olaparib in patients with BRCA1 or BRCA2 mutations and recurrent ovarian cancer: a proof-of-concept trial.** *Lancet* 2010, **376:**245-251.

4. Tutt A, Robson M, Garber JE, Domchek SM, Audeh MW, Weitzel JN, Friedlander M, Arun B, Loman N, Schmutzler RK, Wardley A, Mitchell G, Earl H, Wickens M, Carmichael J: **Oral poly(ADP-ribose) polymerase inhibitor olaparib in patients with BRCA1 or BRCA2 mutations and advanced breast cancer: a proof-of-concept trial.** *Lancet* 2010, **376:**235-244.

5. Gelmon KA, Tischkowitz M, Mackay H, Swenerton K, Robidoux A, Tonkin K, Hirte H, Huntsman D, Clemons M, Gilks B, Yerushalmi R, Macpherson E, Carmichael J, Oza A: **Olaparib in patients with recurrent high-grade serous or poorly differentiated ovarian carcinoma or triple-negative breast cancer: a phase 2, multicentre, open-label, non-randomised study.** *Lancet Oncol* 2011, **12:**852-861.

6. Leichman LP, Cohen SJ, Hochster HS, Messersmith WA, Lenz H, Boman BM, Gold PJ, O'Neill BH, Berlin J, Carmichael J: **A phase II trial to assess the single-agent efficacy and safety of the PARP inhibitor olaparib (O) in previously treated patients (pts) with metastatic, measurable colorectal cancer (mCRC) stratified by microsatellite status (MSs) [abstract].** *Proceedings of the ASCO-NCI-EORTC Annual Meeting on Molecular Markers in Cancer* 2010, Abstract No. 118

7. Lee J, Annunziata CM, Minasian LM, Zujewski J, Prindiville SA, Kotz HL, Squires J, Houston ND, Ji JJ, Yu M, Doroshow JH, Kohn EC: **Phase I study of the PARP inhibitor olaparib (O) in combination with carboplatin (C) in BRCA1/2 mutation carriers with breast (Br) or ovarian (Ov) cancer (Ca) [abstract].** *J Clin Oncol* 2011, **29**(Suppl 15):2520

8. Liu J, Fleming GF, Tolaney SM, Birrer MJ, Penson RT, Berlin ST, Whalen C, Tyburski K, Matijevich K, Kasparian E, Roche M, Lee H, Winer EP, Ivy SP, Matulonis U: **A phase I trial of the PARP inhibitor olaparib (AZD2281) in combination with the antiangiogenic cediranib (AZD2171) in recurrent ovarian or triple-negative breast cancer [abstract].** *J Clin Oncol* 2011, **29**(Suppl 15):5028

9. Kaye S, Kaufman B, Lubinski J, Matulonis U, Gourley C, Karlan B, Taylor D, Wickens M, Carmichael J: **Phase II study of the oral PARP inhibitor olaparib (AZD2281) versus liposomal doxorubicin in ovarian cancer patients with BRCA1 and/or BRCA2 mutations [abstract].** *Ann Oncol* 2010, **21**(Suppl 8):vii304

10. Viswanathan S, Wesolowski R, Layman RM, Alejandra G, Miller B, Chalmers JJ, Bejastani S, Zhao W, Pierluigu G, Cotrill J, Phelps MA, Schaaf LJ, Geyer SM, Hall N, Knopp MV, Shapiro CL, Villalona-Calero MA, Chen A, Grever MR, Ramaswamy B: **A phase I dose-escalation study of ABT-888 (veliparib) in combination with carboplatin in HER2-negative metastatic breast cancer (MBC) [abstract].** *J Clin Oncol* 2011, **29**(Suppl 15):TPS106

11. Zhao W, Duan W, Leon ME, Chen AP, Sofletea G, Thurmond J, Ramaswamy B, O'Malley D, Bekaii-Saab TS, Calero MA: **Targeting fanconi anemia (FA) repair pathway deficiency for treatment with PARP inhibitors [abstract].** *J Clin Oncol* 2010, **28**(Suppl 15):TPS168

12. Middleton M, Friedlander P, Hamid O, Daud A, Plummer R, Schuster R, Qian J, Luo Y, Giranda V, McArthur G: **Efficacy of veliparib (ABT-888) plus temozolomide versus temozolomide alone: a randomized, double-blind, placebo-controlled trial in patients with metastatic melanoma [abstract].** *Programs and abstracts of the 36th European Society for Medical Oncology Congress* 2011, Late breaking abstract 13

13. Pishvaian MJ, Slack R, Witkiewicz A, He AR, Hwang JJ, Hankin A, Dorsch-Vogel K, Kuda D, McAndrew T, Weiner LM, Marshall J, Brody JR: **A phase II study of the PARP inhibitor ABT-888 plus temozolomide in patients with heavily pretreated, metastatic colorectal cancer [abstract].** *J Clin Oncol* 2011, **29**(Suppl 15):3502

14. Pishvaian MJ, Slack R, Witkiewicz A, He AR, Hwang JJ, Hankin A, Ley L, Apte SK, Littman SJ, Weiner LM, Marshall J, Brody JR: **A phase I/II study of the PARP inhibitor, ABT-888 plus 5-fluorouracil and oxaliplatin (modified FOLFOX-6) in patients with metastatic pancreatic cancer [abstract].** *J Clin Oncol* 2011, **29**(Suppl 15):TPS170

15. Andreopoulou E, Chen AP, Zujewski J, Kim M, Hershman DL, Kalinsky K, Cigler T, Vahdat LT, Raptis G, Ramaswamy B, Novik Y, Muggia F, Sparano JA: **Randomized, double-blind, placebo-controlled phase II trial of low-dose metronomic cyclophosphamide alone or in combination with veliparib (ABT-888) in chemotherapy-resistant ER and/or PR-positive, HER2/neu-negative metastatic breast cancer: New York Cancer Consortium trial P8853 [abstract].** *J Clin Oncol* 2011, **29**(Suppl 15):TPS114
