# Supplementary figures and images for: Advances in using PARP inhibitors to treat cancer
Source: BMC Med. 2012 Mar 9;10:25. doi: 10.1186/1741-7015-10-25 (PMC3312820; doi:10.1186/1741-7015-10-25)

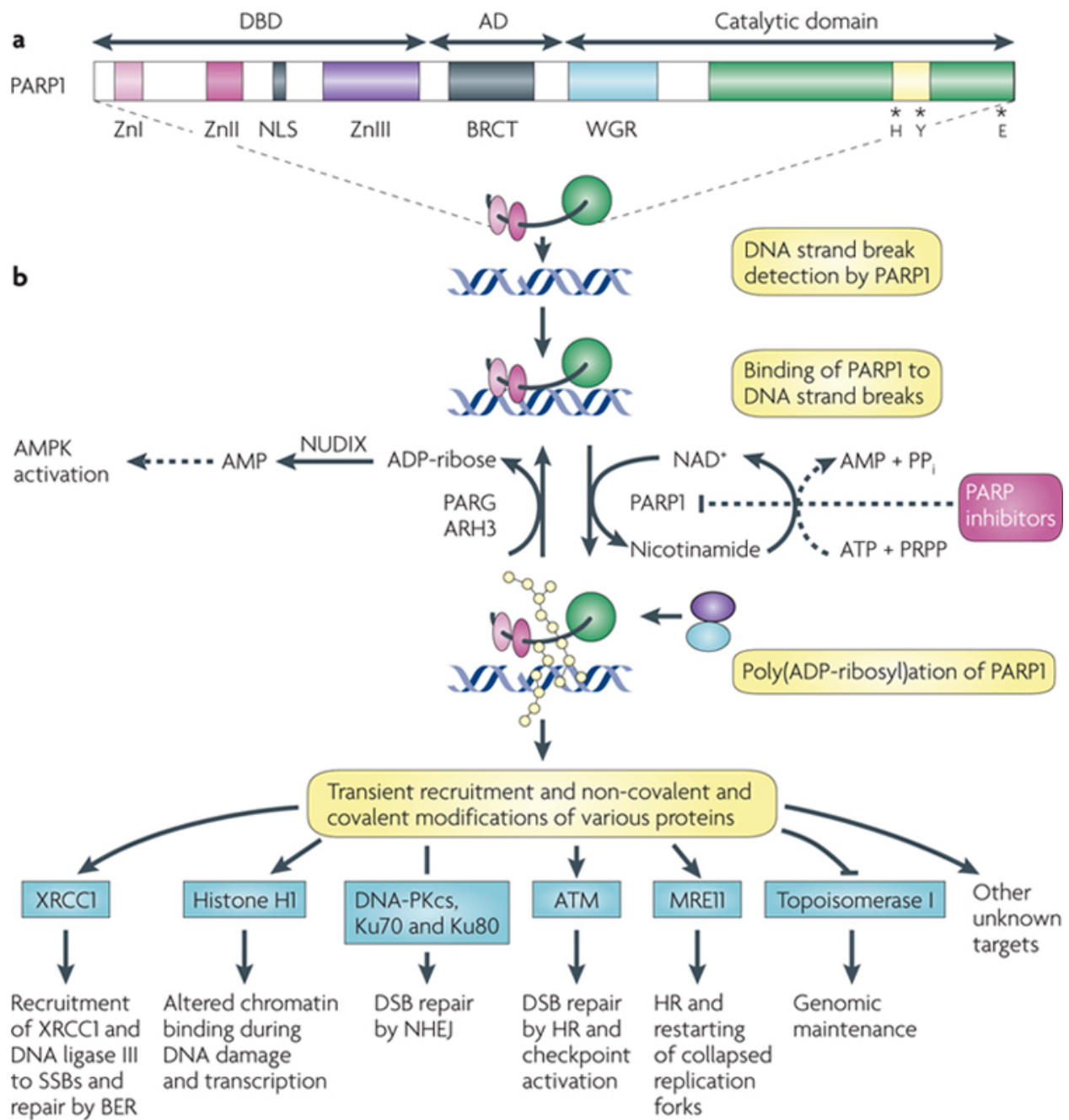

Supplement: Additional File 3 — Structural and functional characteristics of PARP1. A: Poly(ADP-ribose) polymerase 1 (PARP1) is shown with its DNA-binding (DBD), automodification (AD) and catalytic domains. The PARP signature sequence (yellow box within the catalytic domain) comprises the sequence most conserved among PARPs. Crucial residues for nicotinamide adenine dinucleotide (NAD+) binding (histidine; H and tyrosine; Y) and for polymerase activity (glutamic acid; E) are indicated. B: Consequences of PARP1 activation by DNA damage. Although not shown to simplify the scheme, PARP1 is active in a homodimeric form. PARP1 detects DNA damage through its DBD. This activates PARP1 to synthesize poly(ADP) ribose (pADPr; yellow beads) on acceptor proteins, including histones and PARP1. Owing to the dense negative charge of pADPr, PARP1 loses affinity for DNA, allowing the recruitment of repair proteins by pADPr to the damaged DNA (blue and purple circles). Poly(ADP-ribose) glycohydrolase (PARG) and possibly ADP-ribose hydrolase 3 (ARH3) hydrolyse pADPr into ADP-ribose molecules and free pADPr. ADP-ribose is further metabolized by the pyrophosphohydrolase NUDiX enzymes into AMP, raising AMP:ATP ratios, which in turn activate the metabolic sensor AMP-activated protein kinase (AMPK). NAD+ is replenished by the enzymatic conversion of nicotinamide into NAD+ at the expense of phosphoribosylpyrophosphate (PRPP) and ATP. Examples of proteins non-covalently (pADPr-binding proteins) or covalently poly(ADP-ribosyl)ated are shown with the functional consequences of modification. It is important to note that many potential protein acceptors of pADPr remain to be identified owing to the difficulty of purifying pADPr-binding proteins in vivo. PARP inhibitors prevent the synthesis of pADPr and hinder subsequent downstream repair processes, lengthening the lifetime of DNA lesions. ATM, ataxia telangiectasia-mutated; BER, base excision repair; BRCT, BRCA1 carboxy-terminal repeat motif; DNA-PKcs, DNA-protein kinase catal [file 1741-7015-10-25-S3.PDF]
